# 

**Published:** 1951-01

**Authors:** 


					&
PUBLISHED UNDER THE AUSPICES OF THE BRISTOL MEDICO-CHIRURGICAL SOCIETY
THE BRISTOL
MEDICO - CHIRURGICAL
JOURNAL
A Journal oj the Medical Sciences for the
WEST OF ENGLAND
AND SOUTH WALES
Editor:
E. WATSON-WILLIAMS, M.C., M.D., Ch.M., F.R.C.S.
with whom are associated
G. E. F. SUTTON, M.C., M.D., F.^CiPf \
\
J. H. MIDDLEMISS, M.D., D.M.RiD., F.
N. S. CRAIG, M.C., M.D., Assistant Editor.- " ; y/ ' ,^ i
Local Correspondents : IAS 25 mi
Bath?A. D. BATE MAN, oIb.E., F.R.C.S.
Exeter?L. N. JACKSON, a^C., D.M., |
Gloucester?R. F. JARRETT, RTC.r.
Newport?J. L. D. WILLIAMS, M.B.,
Plymouth?C. R. CROFT, D.M., M.R.C.P. Taunton?Dr. M. McALLEY, D.M.R.
Torquay?Dr. A. ROBINSON THOMAS, D.M.R.E.
Truro?Dr. F. D. M. HOCKING, F.R.LC. Yeovil?J. A. VERE NICOLL, F.R.C.S.
BRISTOL J
J. W. ARROWSMITH LTD., QUAY STREET AND SMALL STREET
Vol. LXVHI. No. 245. Price: FOUR SHILLINGS net.
JANUARY ? 1951

				

